# Meeting of Inter-Allied Surgeons

**Published:** 1918-11

**Authors:** 


### Inter-Allied Surgeons.

A most notable surgical meeting was held at Chattanooga, Tenn., October 25-26, which was officially designated as ''The Inter-Allied War Conference of Surgeons.'' In addition to many American surgeons of international reputation, such as Drs. Duncan Eve, George E. Brewer, and Franklin Martin, a number of foreign surgeons were on the program—men like Sir Thomas Myles, of Dublin, former president of the College of Surgeons of Ireland; Prof. Raffaele Bastianelli, of the faculty of the Royal University of Rome; Maj. George Grey Turner, of Newcastle-on-Tyne; Colonel Duval, of the French Reserve Medical Corps, and Col. George Gask, of England.

Col. George Gask, one of England's most distinguished surgeons, in his paper entitled, ''The Early Treatment of Gunshot Wounds,'' said that the most important problems of chest surgery had been solved during the past two years.

''It was formerly the custom to put a man to bed, give him some morphia and a little cough mixture and hope for the best,'' Colonel Gask said. ''As might be expected, the mortality was very great, and in non-fatal cases convalescence was prolonged.''

Colonel Gask told of the indescribable conditions with which doctors had to deal during the first years of the war when soldiers were shipped home to base hospitals still covered with the grime and dirt of the battlefield.

''After the retreat from Mons, we received patients whose wounds were in every imaginable stage of corruption,'' the colonel said. ''I would rather not think about those days. We went through all the experimental stages in dealing with unusual cases.

''A mercury solution failed, and drainage failed. We were blind, we were crazy in our striving for some new form of antiseptic which would kill off all infecting germs over night. Now, we know that it is of primary importance to insure an early mechanical cleansing of wounds.

''We had never intended to utilize casualty clearing stations as surgical hospitals. We soon found out that this was important. Let me describe the successive order of events in caring for men at a great receiving station.

"First, there is the big reception room. Orderlies carry patients to the dressing room where the old, soiled dressings are removed and where the men are inspected. They are either sent back to duty, forwarded to the evacuation wards, or sent to the operating rooms. Shock cases are sent to resusitation wards.

"Scenes in the resuscitation wards are heartrendering. The men are shocked and mangled. One has to grip one's very spirit in order to do effective and serviceable work. It was here that I met some of the first surgeons and doctors of America. I became intimately acquainted with them, studied their work and recognized their merit.

"Over 80 per cent. of all wounds can be closed by primary suture and healed.

"While we no longer fear gas gangrene, we do fear wounds infected with hymelotic streptocus. This is what causes so many deaths."

Colonel Gask then explained from colored charts and pictures the method of treating severe wounds of the body and limbs. One picture showed a forearm which contained twenty-four pieces of shrapnel. The bits of iron and steel were removed and the man recovered.

Colonel Duval laid stress upon the early mechnical cleansing of wounds. He said the doctor must first consider that every wound is infected and that it contains dead tissue. He showed the difference between contamination and infection, stating that infection generally sets in from six to twelve hours after the soldier is taken from the battlefield.

The French surgeon for the second time brought a gasp of admiration from the medicos by his description of block excisions of all dead tissue.

"The ideal operation is the block excision of all dead matter so that only new, live tissue may be joined together," he said. "Dead tissues breed germs and also are responsible for spreading poisonous substances into the system. Therefore, war wounds must be excised to free them from germs, and this must be done at an early period. Goutier, a surgeon at Lille, was among the first to realize this.

"Primary suture is the correct method of treatment if the patient can be kept at the hospital for a considerable length of time—say from eight to ten days. If the doctor knows that a secondary suture will have to follow, he should adapt his technique accordingly. Of course there are surgical impossibilities. It is not always possible to get to the bottom of deep wounds. They must be treated for infection and a secondary suture provided for.

"At one stage of the Somme battle, there were 8,500 casualties of all kinds. The death rate was 6.9 per cent. Total amputations necessary amounted to 1.7 per cent. Four per cent. of the men were permanently incapacitated. In two months 85 per cent. of all the men treated were able to be sent back to duty. These figures bear out the statement that there has truly been a wonderful advance in modern surgery since the first years of the war.

"You American surgeons are fortunate to enter the war at a time when England, France and Italy have added so much to their store of knowledge. You are lucky in that you will be able to save so many lives when we, in our first experimental stages were unable to do so."

Professor Bastianelli pointed out that it was poor surgery for a man simply to content himself with the knowledge that the stump of an amputated arm or leg would never bother a soldier again. He said that it was the doctor's duty to so conduct his operation that the soldier would be of some use in the world when resuming the normal activities of civil life.

"In 1896 Vanghetti, the Italian surgeon, discovered that artificial fingers may be moved by merely flexing the muscles. The mind controls the muscles.

"In performing the amputation, do not trust the stray muscles to take care of themselves. They should be joined together attached by the loop, or knob system, so that later they may be attached to artificial fingers and joints."

The speaker then explained how muscles may be "hitched" together so that they will co-ordinate and function. To the audience, his dissertation of this remarkable operation was comparable to the expert manner in which a skilled electrician connects the positive and negative electrodes of a storage battery so that the completed circuit thus formed will produce heat and light.

Going still deeper into the intricacies of surgery, Colonel Duval told how it was possible to outfit a man with an efficient set of fingers, even after the hand had been corrupted by infection. His description of "splicing" muscles to give them a more perfect pulling power reminded his hearers of the way a sailor will splice several thin strands of rope into a strong cable, capable of a resistance greater than the ununited effectiveness of the strands.

The final stages of this operating do not take place until the patient is well on the road to recovery, he stated. Then, the muscles are so united that they will lie in canals and will respond readily to the flexing of the muscles of the arm. The lower ends of these muscles actually protrude out from skin flaps and are joined to artificial fingers.

Colonel Brewer gave a dramatic description of a bombardment of an American base hospital seventeen kilometers behind the French lines near Chalons. He made known for the first time that the German attacks on Rheims, Chalons and Chateau Thierry were known to the French secret service in advance and that the Fourth French army had been able to make partial preparations to guard against them. He told in detail the newest methods for the treatment of shell shock cases.

"America can be credited with supplying a general outline for the treatment of shell shock," he said. "When big shells are bursting all around, the amount of genuine, traumatic shell shock is very great.

"Severe wounds, exhaustion, cold and hunger also induce shock. Right after the early stages of the battle of Chateau Thierry, I came upon a group of American soldiers who were lying upon the ground in an apparent state of exhaustion. Their eyes were sunken, they had a deadly pallor and their general appearance was that of impending death.

"'We've been for four days without food or sleep,' they told me. 'We've been through hell and we haven't had any water.' Now these men, only slightly wounded, were in need of treatment just as much as if they had a leg or an arm shot away.

"I suggested, in my report to the surgeon-general, that we ought to have a special shock corps for the treatment of these cases. Blood transfusion and appliances for heating form part of the treatment. The result is that now every unit has a special 'shock team' or corps, whose duty it is to look after men who have been incapacitated in this way.

"Surgeons no longer have to worry about cases with shock symptoms that have passed through their hands. They know that another department of the hospital is giving the men the best treatment science affords.

"The shock teams in the hospital in which I worked back of the British lines before the big push on Passchendale consisted of two doctors, two nurses and two orderlies. They had special electric stoves which were placed under the beds so that the temperatures under the blankets would rise to over 100 degrees. The patients were also treated with a 6 per cent salt solution to increase the blood pressure.

"We were ready when the Germans opened fire in their big drive forward. Though seventeen kilometers away, the roar of the big guns was continuous. About 1 a. m. the first casualties began to come in. There were six operating theaters and as fast as the ambulances rushed up the men were brought inside. We had accommodations for 1,500 patients. Let me explain that ours was a movable hospital, consisting of collapsible tents, which could be taken down in three and one-half hours and put up in eight.

"By previous arrangement with the French ranking surgeon, we were prepared to evacuate upon short notice and fall back to a position in the rear, which had also been agreed upon.

"Right in the midst of the rush there was a piercing screech. A 305 m. shell whistled overhead. I could have sworn that it struck just outside of the building, but in reality it burst half a mile away. These shells now began to come over at four or five-minute intervals, and I noticed that every one was striking nearer our buildings.

"Finally one broke about the length of this building away (eighty feet). Then several more landed with a thud. A hay stack caught fire and we could see the blaze plainly through the windows. Right here I want to say a word for the way those English nurses bore up under the terrible strain and excitement of the bombardment. Each knew that the boche was sending over a creeping barrage in the hope of catching us, yet they and the orderlies who worked with them went about their tasks with a composure that was wonderful.

"Operations went on with those terrible shells plowing up the ground only a few feet away. Nearer and nearer came the barrage, and at last the boche made a direct hit on one of the outlying buildings. We had built huge abiis, or dugouts, capable of housing 175 to 200 patients not far away. Patients and nurses were rushed to within their semi-protecting walls. Not a man or woman lost his head during the hasty transfer.

"Within the next half hour there were six more direct hits on hospital buildings. Only the first resulted in casualties. Finally the order was given to fall back. All equipment was loaded on thirty-eight trucks and we retired in good condition.

"The hospital was cited by General Pershing for that night's work."

Among the new wonders of surgery demonstrated to the assembled doctors was the use of a rubber balloon employed as a stoppage to the chest cavity after a major operation. Prof. Raffaille Bastianelli, of the University of Rome, showed how this bag might be inserted into the chest cavity and then inflated to prevent infected air from entering the chest walls. According to Prof. Bastianelli, this new device may be used to patch up a wounded man after the fashion that an automobilist uses a rubber plug to patch a leaking tire.

"I have removed the human lung from the chest cavity with forceps, tied its bleeding blood vessels, cleansed its outer surface, and while still holding it in my hands and manipulating it as you would a handkerchief, I have run thin pieces of gauze up its tracts. Feeling my way carefully along its walls I have removed a bullet or shell fragment. Then, after suturing the aperture, I have placed the respiratory organ back into the cavity of the chest. In two-thirds of the cases upon which I have operated, the patient lived."

At this meeting, which was the first congress of inter-allied surgeons that has been held in America since the war began, there were 1,200 medical officers of the American Army present. They were from all parts of the country, and the information which they gathered will be of great value to the profession, not only during the war, but after it has ended. It was indeed a great privilege to American medical men to hear such discussions from the most able surgeons who have seen long service at the front.

